# Emotional labor and burnout among healthcare workers in Korea: occupation-specific moderated mediation through job satisfaction (a cross-sectional secondary analysis)

**DOI:** 10.1186/s12913-026-14166-1

**Published:** 2026-02-07

**Authors:** Yo-Han Seo, Eun-Taek Hong

**Affiliations:** Department of Laboratory Medicine, Gwangju Veterans Hospital, Korea Veterans Health Service, Cheomdanwolbong-ro, Gwangsan-gu, Gwangju, Republic of Korea

**Keywords:** Burnout, Emotional labor, Healthcare workers, Job satisfaction, Moderated mediation, Structural equation modelling

## Abstract

**Background:**

Evidence links emotional labor to burnout among hospital workers, yet most studies focus on nurses or aggregate diverse allied health occupations into a single group. This cross-sectional secondary analysis examined whether job satisfaction mediates the association between emotional labor and burnout, and whether the mediation pathway differs by occupation among hospital-based healthcare workers.

**Methods:**

We analyzed data from a standardized self-administered survey conducted in July 2022 across six hospitals under the Korea Veterans Health Service. Of 570 eligible healthcare workers, 290 responded (response rate 50.9%), including clinical laboratory scientists, radiologic technologists, physical therapists, and dental hygienists. Emotional labor, job satisfaction, and burnout were assessed using validated questionnaires. We applied generalized structural equation modeling to test an occupation-specific moderated mediation model (emotional labor → job satisfaction → burnout), adjusting for age, sex, employment status, weekly working hours, self-rated health status, and hospital.

**Results:**

Dental hygienists showed the highest mean emotional labor and burnout levels. In the overall mediation model, higher emotional labor was associated with lower job satisfaction (a = − 0.765; 95% CI − 0.857 to − 0.674) and higher burnout both directly (c′ = 0.340; 95% CI 0.261 to 0.420) and indirectly via job satisfaction (indirect effect = 0.096; 95% CI 0.050 to 0.143), corresponding to 22.1% mediation of the total effect (total effect = 0.436; 95% CI 0.357 to 0.516). In occupation-specific models, the indirect effect through job satisfaction was significant for clinical laboratory scientists and radiologic technologists, whereas the direct effect of emotional labor on burnout remained significant across all four occupations.

**Conclusion:**

Emotional labor was positively associated with burnout among healthcare workers, and job satisfaction accounted for a meaningful portion of this association. The mediation pathway differed by occupation, suggesting that burnout mitigation strategies may benefit from occupation-tailored approaches, with particular attention to strengthening job satisfaction where mediation is evident.

**Supplementary Information:**

The online version contains supplementary material available at 10.1186/s12913-026-14166-1.

## Background

Hospitals increasingly emphasise patient-centred service and responsiveness to patients’ needs, which can intensify interpersonal and emotional demands on frontline healthcare workers [1, 2]. In this context, emotional labor refers to managing emotional expressions to comply with organisational “display rules,” and is conceptually distinct from physical or cognitive labor [3]. Contemporary frameworks further describe emotional labor as a form of emotion regulation at work, with heterogeneous implications depending on occupational role and the strategies used (e.g., surface vs. deep acting) [2]. In Korea, government-briefing summaries of national occupational analyses have also highlighted that service-facing roles—including several healthcare-related occupations—are frequently characterised by high emotional-demand profiles [4].

Burnout is commonly understood as a work-related syndrome arising from prolonged job stress and was first described in health-service contexts [5]. In healthcare settings, burnout is frequently conceptualised as emotional exhaustion, mental distancing/depersonalisation (cynicism), and reduced personal accomplishment [6]. Burnout can undermine individual well-being and is associated with reduced job performance, safety concerns, and turnover intention, ultimately threatening continuity and quality of care [7, 8].

Accumulating evidence indicates that emotional labor is meaningfully associated with impaired well-being and adverse job attitudes in healthcare workers [2, 9, 10]. Job satisfaction is a plausible explanatory pathway linking emotional demands to burnout-related outcomes; in job demands–resources (JD-R) theory, emotionally demanding working conditions function as job demands that elevate strain, while resources and positive job attitudes may mitigate burnout risk [11, 12]. Accordingly, a statistical mediation model in which emotional labor is associated with lower job satisfaction, and lower job satisfaction is associated with higher burnout, is theoretically coherent and empirically testable—while recognising that cross-sectional mediation should be interpreted as an association-consistent pathway rather than causal proof.

Despite this, prior hospital-based research has often focused on nurses [1, 9, 10] or has aggregated allied health professionals into a single occupational category, potentially obscuring occupation-specific patterns [2]. International evidence also indicates that job satisfaction and perceived stress are related among radiology professionals [13]. Exis ing studies in Korea suggest that emotional labor, job satisfaction, and burnout are interrelated among clinical laboratory scientists/medical technologists [14, 15], tradiologic technologists/radiographers [16, 17], physical therapists [18–20], and dental hygienists [21, 22]. Building on our previous report using the same K-VHS survey dataset [15], the present cross-sectional secondary analysis explicitly tests an occupation-specific moderated mediation model using structural equation modelling (SEM): we examine whether job satisfaction statistically mediates the association between emotional labor and burnout and whether the magnitude of these pathways differs across occupations (Occupation: 1 = Clinical laboratory scientist, 2 = Radiologic technologist, 3 = Physical therapist, 4 = Dental hygienist).Therefore, this study aims to (i) quantify the direct association between emotional labor and burnout, (ii) estimate the indirect (mediated) association through job satisfaction, and (iii) evaluate whether these paths vary by occupation. We hypothesised that higher emotional labor would be associated with higher burnout both directly and indirectly via lower job satisfaction, and that the indirect effect would differ across occupational groups.

## Materials and methods

### Participants

This study included 570 healthcare workers from six hospitals under the Korea Veterans Health Service (VHS Medical Center, Gwangju, Busan, Daegu, Daejeon, and Incheon Veterans Hospitals): clinical laboratory scientists, radiologic technologists, physical therapists, and dental hygienists. The required sample size was estimated as 280 using G*Power (version 3.1.9.7). Due to the COVID-19 pandemic and the geographic distribution of participating hospitals, the survey was administered online (Google Forms) between July 1 and July 31, 2022. A total of 290 participants completed the questionnaire (response rate 50.9%). Before accessing the questionnaire, participants reviewed an information sheet describing the study purpose, voluntary participation, anonymity/confidentiality, and data handling. Participants provided informed consent electronically by selecting an ‘I agree’ option, after which the questionnaire became accessible. No personally identifying information was collected. The study protocol was approved by the Institutional Review Board of the Gwangju Veterans Hospital (IRB no.: human 2022-12-6).

### Measures

The questionnaire consisted of (i) items on sociodemographic and work-related characteristics, including subjective health status and job-related considerations, and (ii) standardized multi-item scales assessing emotional labor, job satisfaction, and burnout. Items requiring reverse scoring were recoded prior to analysis. For transparency and reproducibility, the full questionnaire (English version) is provided as a supplementary file (Additional file X).

#### Sociodemographic and work-related characteristics, and subjective health status

Covariates included sex, age group, marital status, body mass index (BMI), exercise habit, hobbies, educational attainment, religion, affiliated hospital, occupation, customer-facing duties, work pattern, length of service, job position, monthly income, commute time, and subjective health status.

Age was categorized as 20s, 30s, 40s, 50s, and ≥ 60 years. BMI was calculated from self-reported height and weight and categorized using Korean clinical guideline cut-points (underweight < 18.5 kg/m², normal 18.5–22.9 kg/m², and overweight ≥ 23.0 kg/m²) [23]. Subjective health status was assessed on a five-level scale (very poor, poor, moderate, good, very good).

Occupation was coded as: Occupation (1 = Clinical laboratory scientist, 2 = Radiologic technologist, 3 = Physical therapist, 4 = Dental hygienist).

#### Job choice and job-change considerations

Participants were asked to indicate the primary factor considered when choosing their current job and when considering a job change. Response options were: pay; working environment and welfare; company size and growth opportunities; education system; transportation convenience; and other.

#### Emotional labor

Emotional labor was assessed using a 24-item Korean emotional labor instrument covering five domains: emotional demand and regulation (5 items), overload and conflict in customer service (3 items), emotional disharmony and hurt (6 items), organisational surveillance and monitoring (3 items), and lack of a supportive/protective system (7 items) [24]. Items were rated on a Likert-type scale, with higher scores indicating greater emotional labor. Internal consistency in the present study was acceptable (Cronbach’s α = 0.76).

#### Job satisfaction

Job satisfaction was measured using a 37-item instrument based on the Job Descriptive Index framework and a Korean adaptation used in healthcare settings [25]. The scale covers seven domains: the work itself (5 items), pay (7 items), supervision (7 items), promotion (5 items), coworkers (5 items), organisation (5 items), and job change (4 items). Items were rated on a Likert-type scale, with higher scores indicating higher job satisfaction. Internal consistency was good (Cronbach’s α = 0.85).

#### Burnout

Burnout was measured using the Maslach Burnout Inventory (MBI) [6]. The MBI consists of 22 items across three subscales: emotional exhaustion (9 items), depersonalisation (5 items), and reduced personal accomplishment (8 items). Items were rated on a 7-point Likert-type scale (0 = never to 6 = daily); higher summed scores indicate higher burnout (Cronbach’s alpha = 0.89 in this study). The MBI has been used in Korean occupational research [26].

### Statistical analysis

Analyses were conducted using Stata/MP 14.1 (StataCorp LLC, College Station, TX, USA). Descriptive statistics were used to summarise participant characteristics and key study variables; group differences across occupations were examined using χ² tests (or Fisher’s exact tests where appropriate) for categorical variables and one-way ANOVA (or non-parametric alternatives where appropriate) for continuous variables (Table 1).

**Table 1:** Sociodemographic and work-related characteristics of the study participants by occupation (*N* = 290)

Variables	Clinical laboratory scientists (*n* = 89)	Radiologic technologists (*n* = 67)	Physical therapists (*n* = 82)	Dental hygienists (*n* = 52)	Total (*N*)	*p*
Sex						< 0.001
Male	36(40.4)	51(76.1)	57(69.5)	3(5.8)	147	
Female	53(59.6)	16(23.9)	25(30.5)	49(94.2)	143	
Age (year)						0.212
20–29	16(18.0)	12(17.9)	18(22.0)	10(19.2)	56	
30–39	38(42.7)	29(43.3)	38(46.3)	25(48.1)	130	
40–49	17(19.1)	11(16.4)	18(22.0)	15(28.9)	61	
50–60	18(20.2)	15(22.4)	8(9.7)	2(3.8)	43	
Marital status						0.671
Unmarried	44(49.4)	26(38.8)	36(43.9)	21(40.4)	127	
Married	45(50.6)	41(61.2)	46(56.1)	31(59.6)	163	
BMI						0.001
Underweight (< 18.5)	4(4.5)	6(9.0)	5(6.1)	5(9.6)	20	
Normal (18.5–22.9)	47(52.8)	19(28.3)	33(40.2)	29(55.8)	128	
Overweight (≥ 23)	38(42.7)	42(62.7)	44(53.7))	18(34.6)	142	
Hobbies and exercise						0.069
No	32(36.0)	16(23.9)	17(20.7)	19(36.5)	84	
Yes	57(64.0)	51(76.1)	65(79.3)	33(63.5)	206	
Educational level						0.001
Junior college	26(29.2)	23(34.3)	11(13.4)	20(38.5)	80	
University	52(58.4)	41(61.2)	56(68.3)	28(53.8)	177	
Graduate school	11(12.4)	3(4.5)	15(18.3)	4(7.7)	33	
Religion						0.743
No	30(33.7)	28(41.8)	32(39.0)	21(40.4)	111	
Yes	59(66.3)	39(58.2)	50(61.0)	31(59.6)	179	
Affiliated veterans hospital						0.598
Central	26(29.3)	24(35.8)	15(18.3)	18(34.6)	83	
Gwangju	18(20.2)	14(20.9)	29(35.4)	9(17.3)	70	
Busan	14(15.7)	10(14.9)	13(15.9)	8(15.4)	45	
Daegu	16(18.0)	8(11.9)	6(7.3)	7(13.5)	37	
Daejeon	10(11.2)	4(6.0)	6(7.3)	8(15.4)	28	
Incheon	5(5.6)	7(10.5)	13(15.8)	2(3.8)	27	
Customer service						< 0.001
No	45(50.6)	2(3.0)	1(1.2)	3(5.8)	51	
Yes	44(49.4)	65(97.0)	81(98.8)	49(94.2)	239	
Work pattern						< 0.001
Day work	64(71.9)	51(76.1)	82(100.0)	51(98.1)	248	
Day and night shifts	25(28.1)	16(23.9)	0	1(1.9)	42	
Service period (year)						< 0.001
<2	8(9.0)	7(10.5)	33(40.2)	7(!3.5)	55	
2–4	16(18.0)	20(29.9)	15(18.3)	12(23.1)	63	
5–9	24(27.0)	9(13.4)	12(14.6)	8(15.4)	53	
10–19	14(15.7)	15(22.3)	13(15.9)	19(36.5)	61	
≥20	27(30.3)	16(23.9)	9(11.0)	6(11.5)	58	
Position						0.087
Staff	30(33.7)	24(35.8)	45(54.9)	25(48.1)	124	
Assistant manager	40(44.9)	26(38.7)	23(28.0)	20(38.5)	109	
Senior staff	9(10.1)	7(10.5)	5(6.1)	4(7.7)	25	
Manager	7(7.9)	6(9.0)	6(7.3)	3(5.8)	16	
General manager	3(3.4)	4(6.0)	3(3.7)	0	10	
Pay (￦10.000/Month)						0.001
〈200	2(2.3)	2(3.0)	1(1.2)	2(3.8)	7	
200–299	10(11.2)	10(14.9)	38(46.3)	17(32.7)	75	
300–399	44(49.4)	34(50.6)	25(30.5)	25(48.1)	128	
400–499	26(29.2)	11(16.5)	11(13.4)	7(13.5)	55	
500–599	3(3.4)	7(10.5)	4(4.9)	0	14	
600≤	4(4.5)	3(4.5)	3(3.7)	1(1.9)	11	
Subjective health status						0.142
Very poor	4(4.5)	3(4.5)	4(4.9)	3(5.8)	14	
Poor	16(18.0)	14(20.9)	18(22.0)	9(17.3)	57	
Moderate	35(39.3)	28(41.8)	38(46.3)	14(26.9)	115	
Good	26(29.2)	20(29.8)	16(19.5)	16(30.8)	78	
Very good	8(9.0)	2(3.0)	6(7.3)	10(19.2)	26	
Commute time						0.201
<30 min	48(53.9)	37(55.2)	49(59.8)	34(65.4)	168	
<1 h	27(30.3)	24(35.8)	23(28.0)	16(30.8)	90	
<1 h 30 min	12(13.5)	2(3.0)	10(12.2)	2(3.8)	26	
<2 h	0	2(3.0)	0	0	2	
≥2 h	2(2.3)	2(3.0)	0	0	4	
Criteria for job selection						0.115
Pay	26(29.2)	12(17.9)	14(17.1)	17(32.7)	69	
Work environment and welfare	44(49.4)	40(59.7)	61(74.3)	32(61.5)	177	
Company size and growth opportunities	11(12.4)	8(11.9)	4(4.9)	1(1.9)	24	
Education system	0	0	0	0	0	
Transportation convenience	2(2.3)	4(6.0)	0	1(1.9)	7	
Other	6(6.7)	3(4.5)	3(3.7)	1(1.9)	13	
Criteria for a job change						0.358
Pay	39(43.8)	30(44.8)	37(45.1)	13(25.0)	119	
Work environment and welfare	41(46.1)	28(41.8)	39(47.6)	35(67.3)	143	
Company size and growth opportunities	6(6.7)	7(10.4)	5(6.1)	2(3.8)	20	
Education system	1(1.1)	0	0	0	1	
Transportation convenience	2(2.3)	1(1.5)	0	1(1.9)	4	
Other	0	1(1.5)	1(1.2)	1(1.9)	3	
Emotional labor	79.79 ± 11.86	79.85 ± 13.01	77.94 ± 14.07	85.56 ± 11.73	290	0.009
Job satisfaction	114.98 ± 22.55	117.18 ± 21.74	120.95 ± 19.61	112.90 ± 22.14	290	0.146
Burnout	61.03 ± 11.42	60.25 ± 12.25	57.22 ± 11.21	66.23 ± 11.39	290	< 0.001

Data are presented as n (%) unless otherwise indicated. Emotional labor, job satisfaction, and burnout are presented as mean ± SD

*p*-values for categorical variables were calculated using the chi-square test or Fisher’s exact test, as appropriate. p-values for continuous variables were calculated using one-way analysis of variance (ANOVA)

Abbreviations: BMI, body mass index

To provide a descriptive assessment of covariates associated with burnout, we additionally fitted a multiple linear regression model with burnout score as a continuous outcome in the full sample (Table S2). The model included emotional labor and job satisfaction as key predictors and adjusted for prespecified sociodemographic and job-related covariates. Regression results are reported as unstandardised coefficients with 95% confidence intervals; the full model output is provided in Table S2.

To test whether associations differed by occupation, we fitted a pooled interaction model that included interaction terms between occupation and key predictors (emotional labor and job satisfaction); where interactions were observed, we reported occupation-specific marginal effects (Table 2 and Table S1).

**Table 2:** Main effects of emotional labor and job satisfaction on burnout in the interaction model

Term	β	Robust SE	*p*	95% CI
Emotional labor (Emotional labor (mean-centered))	0.26	0.10	0.010	0.06 to 0.45
Job satisfaction (Job satisfaction (mean-centered))	-0.13	0.05	0.012	-0.24 to -0.03

Overall interaction tests: Emotional labor×occupation *p* = 0.28; Job satisfaction×occupation *p* = 0.18

Occupation-specific direct, indirect, and total effects of emotional labor on burnout through job satisfaction (moderated mediation model)

Occupation (1 = Clinical laboratory scientist, 2 = Radiologic technologist, 3 = Physical therapist, 4 = Dental hygienist)

We then evaluated whether job satisfaction statistically accounted for part of the association between emotional labor and burnout using a structural equation modelling framework. We estimated direct, indirect, and total associations in the overall sample (Table 3) and in an occupation-specific moderated mediation model (Table 4). All models were interpreted as associative rather than causal given the cross-sectional design. Model evaluation for the moderated mediation SEM estimated using gsem with robust standard errors was summarised using log-likelihood and information criteria (AIC/BIC). Statistical significance was defined as *p* < 0.05 (two-sided).

**Table 3:** Direct, indirect, and total effects of emotional labor on burnout through job satisfaction

Effect	Estimate	SE	*p*	95% CI	Method
Indirect (a×b)	0.10	0.02	< 0.001	0.05 to 0.14	nlcom (delta)
Direct (c′)	0.34	0.05	< 0.001	0.25 to 0.43	nlcom (delta)
Total	0.44	0.04	< 0.001	0.36 to 0.52	nlcom (delta)
Indirect (a×b)	0.10	0.03	0.001	0.04 to 0.15	bootstrap
Direct (c′)	0.34	0.05	< 0.001	0.24 to 0.44	bootstrap
Total	0.44	0.05	< 0.001	0.34 to 0.53	bootstrap

Proportion mediated ≈ 22.10% (indirect/total)

**Table 4:** Occupation-specific direct, indirect, and total effects of emotional labor on burnout through job satisfaction (moderated mediation model)

Occupation	*N*	a: Emotional labor → Job satisfaction	b: Job satisfaction → Burnout	Direct: Emotional labor → Burnout	Indirect: a × b	Total: direct + indirect
Clinical laboratory scientist	89	-0.64 [-0.95, -0.32]; *p* < 0.00	-0.15 [-0.24, -0.06]; *p* = 0.00	0.26 [0.11, 0.42]; *p* = 0.00	0.10 [0.01, 0.18]; *p* = 0.02	0.36 [0.21, 0.51]; *p* < 0.00
Radiologic technologist	67	-0.91 [-1.16, -0.67]; *p* < 0.00	-0.24 [-0.37, -0.12]; *p* < 0.00	0.29 [0.10, 0.49]; *p* = 0.00	0.22 [0.08, 0.36]; *p* = 0.00	0.52 [0.36, 0.67]; *p* < 0.00
Physical therapist	82	-0.71 [-0.95, -0.46]; *p* < 0.00	-0.09 [-0.19, 0.01]; *p* = 0.07	0.37 [0.24, 0.50]; *p* < 0.00	0.07 [-0.01, 0.14]; *p* = 0.08	0.43 [0.32, 0.54]; *p* < 0.00
Dental hygienist	52	-0.91 [-1.40, -0.43]; *p* < 0.00	-0.05 [-0.20, 0.10]; *p* = 0.53	0.49 [0.21, 0.77]; *p* = 0.00	0.05 [-0.09, 0.18]; *p* = 0.53	0.53 [0.32, 0.75]; *p* < 0.00

Model: gsem with interaction terms (occupation moderates a, b, and direct paths). Covariates (SEM-CORE): sex, age, employ, weekly working hours, poorhealth, area. Values are coefficient [95% CI]; p-value

Emotional labor was mean-centered

Job satisfaction and Burnout (total score) were modeled as continuous outcomes with Gaussian family and identity link

Robust standard errors were used

Model evaluation (gsem): *N* = 290; log likelihood=-2197.927; AIC = 4505.854; BIC = 4707.697

## Results

### Sociodemographic and work-related characteristics by occupation

Among 290 participants, sociodemographic and work-related characteristics differed across occupational groups for sex (*p* < 0.001), BMI category (*p* = 0.001), educational level (*p* = 0.001), customer-facing duties (*p* < 0.001), work pattern (*p* < 0.001), length of service (*p* < 0.001), and monthly income (*p =* 0.001). No evidence of between-group differences was observed for age group (*p =* 0.212), marital status (*p =* 0.671), hobbies/exercise (*p =* 0.069), religion (*p =* 0.743), affiliated hospital (*p =* 0.598), job position (*p =* 0.087), subjective health status (*p =* 0.142), commute time (*p =* 0.201), or job selection/change considerations (*p =* 0.115 and *p =* 0.358, respectively) (Table 1).

Mean emotional labor scores (range 24–120) differed by occupation (*p =* 0.009), with the highest mean among dental hygienists (85.56 ± 11.73), followed by radiologic technologists (79.85 ± 13.01), clinical laboratory scientists (79.79 ± 11.86), and physical therapists (77.94 ± 14.07). Mean job satisfaction scores (range 37–185) did not differ by occupation (*p =* 0.146), although physical therapists showed the highest mean (120.95 ± 19.61) and dental hygienists the lowest (112.90 ± 22.14). Burnout scores (range 22–110) differed across groups (*p <* 0.001), with the highest mean among dental hygienists (66.23 ± 11.39) and the lowest among physical therapists (57.22 ± 11.21) (Table 1).

### Main effects and occupation-specific marginal effects in the interaction model

In the pooled interaction model, higher emotional labor was positively associated with higher burnout (β = 0.2555, robust SE = 0.0984, *p =* 0.010; 95% CI 0.0617 to 0.4494), whereas higher job satisfaction was inversely associated with burnout (β=−0.1346, robust SE = 0.0530, *p =* 0.012; 95% CI − 0.2389 to − 0.0303) (Table 2).

Occupation-specific marginal effects showed heterogeneity in these associations (Table S1). The marginal association between emotional labor and burnout was largest among dental hygienists (dydx = 0.5516, *p <* 0.001) and physical therapists (dydx = 0.3661, *p <* 0.001), followed by clinical laboratory scientists (dydx = 0.2555, *p =* 0.010); it was weaker and not statistically supported among radiologic technologists (dydx = 0.2327, *p =* 0.061). In contrast, the inverse marginal association between job satisfaction and burnout was strongest among radiologic technologists (dydx = − 0.2457, *p =* 0.001) and clinical laboratory scientists (dydx = − 0.1346, *p =* 0.012), and weaker among physical therapists (dydx = − 0.1093, *p =* 0.059) and dental hygienists (dydx = − 0.0239, *p =* 0.769) (Table S1).

### Mediation of the emotional labor–burnout association through job satisfaction

**Fig. 1:**
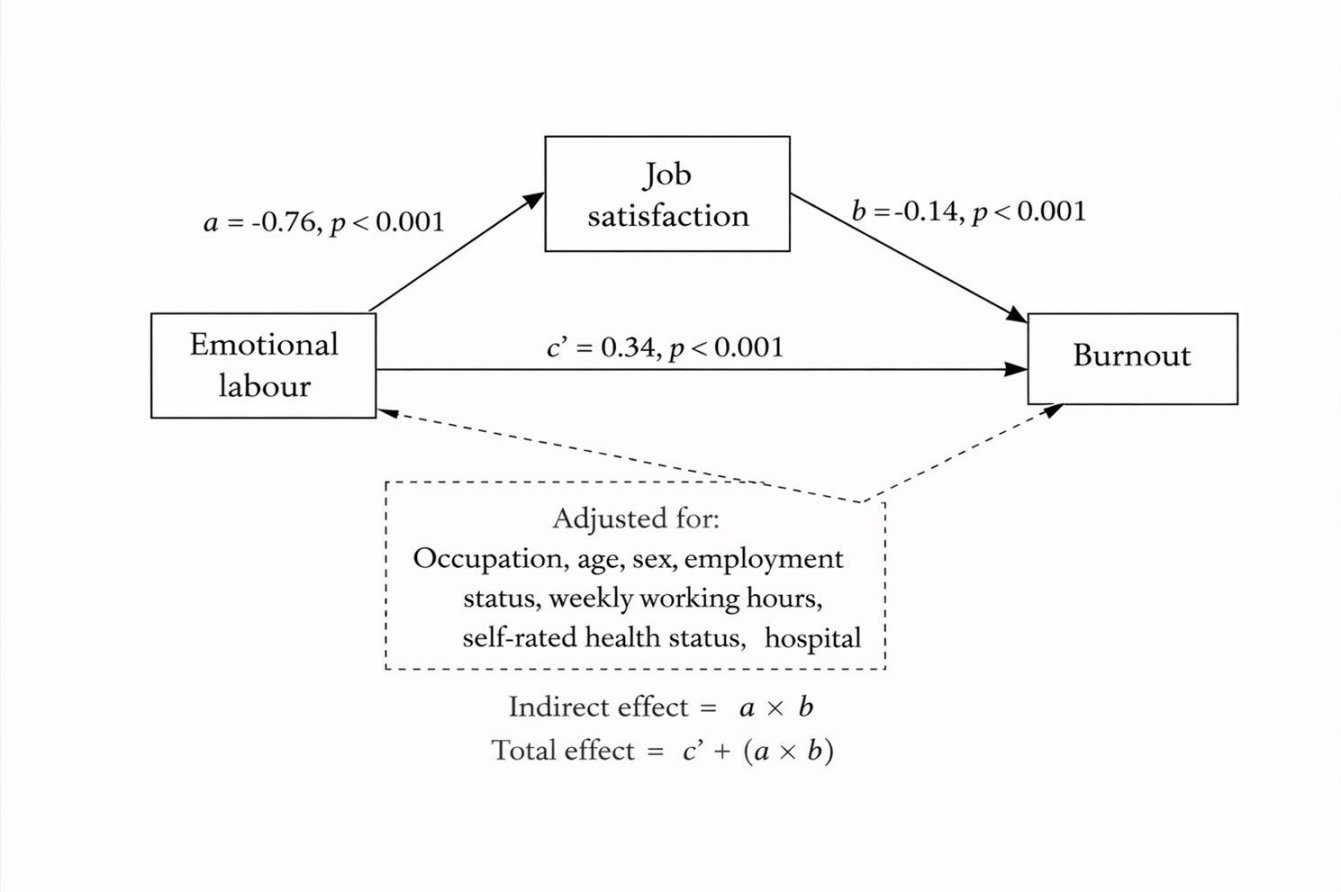
Structural equation model of emotional labor, job satisfaction, and burnout

In the mediation model (Fig. 1), emotional labor was inversely associated with job satisfaction (path a), and job satisfaction was inversely associated with burnout (path b), yielding a positive indirect association of emotional labor with burnout through job satisfaction. Using bootstrap inference, the indirect association (a×b) was 0.0962 (SE = 0.0298, *p =* 0.001; 95% CI 0.0379 to 0.1546). The direct association (c′) remained positive (0.3399, SE = 0.0532, *p <* 0.001; 95% CI 0.2355 to 0.4442), and the total association was 0.4361 (SE = 0.0484, *p <* 0.001; 95% CI 0.3413 to 0.5309) (Table 3). The estimated indirect component accounted for approximately 22% of the total association.

### Occupation-specific moderated mediation (structural equation modelling)

Occupation-specific moderated mediation results are presented in Table 4. The indirect association through job satisfaction was statistically supported among clinical laboratory scientists (indirect = 0.096, *p =* 0.023) and radiologic technologists (indirect = 0.222, *p =* 0.002), but not among physical therapists (indirect = 0.066, *p =* 0.084) or dental hygienists (indirect = 0.045, *p =* 0.527). Across all occupations, the direct association between emotional labor and burnout remained positive (all *P* ≤ 0.003). The total association was largest among dental hygienists (total = 0.534, *p <* 0.001) and radiologic technologists (total = 0.516, *p <* 0.001).

### Supplementary occupation-stratified regression analyses

As a supplementary analysis, a fully adjusted multiple linear regression model in the full sample (Table S2) showed that higher emotional labor was associated with higher burnout (B = 0.362, *p* < 0.001) and higher job satisfaction was associated with lower burnout (B = − 0.124, *p* < 0.001). Several covariates were also associated with burnout in this model, including sex (Female: B = 3.241, *p* = 0.008), age group (50–60: B = − 7.649, *p* = 0.002), BMI (overweight: B = 4.087, *p* = 0.014), employment status (permanent contract: B = − 18.779, *p* = 0.029), and length of service (5–9 years: B = 5.295, *p* = 0.026)(Table S2).

## Discussion

In this cross-sectional secondary analysis of healthcare workers in the Korea Veterans Health Service (K-VHS), we found that the emotional labor–burnout relationship varied by occupation rather than following a single uniform pattern. In pooled interaction analyses, higher emotional labor was associated with higher burnout and higher job satisfaction was associated with lower burnout (Table 2). Occupation-specific marginal effects further indicated meaningful heterogeneity: the marginal association between emotional labor and burnout was strongest among dental hygienists and physical therapists, whereas the inverse association between job satisfaction and burnout was strongest among radiologic technologists and clinical laboratory scientists (Table S1).

In the structural equation modelling (SEM) framework, emotional labor was inversely associated with job satisfaction (path a), and job satisfaction was inversely associated with burnout (path b), yielding a statistically supported indirect association of emotional labor with burnout via job satisfaction in the overall sample (Fig. 1; Table 3). Notably, moderated mediation results suggested that this indirect pathway through job satisfaction was evident among clinical laboratory scientists and radiologic technologists, but not among physical therapists or dental hygienists (Table 4). This secondary analysis extends previous regression-based findings by modeling the indirect pathway via job satisfaction and quantifying occupation-specific differences. The moderated mediation model was evaluated using log-likelihood and information criteria (AIC/BIC), and interpretation focused on the plausibility and consistency of occupation-specific pathway estimates and indirect effects. Taken together, these findings indicate that occupation-tailored strategies may be more effective than a single approach applied uniformly across the workforce.

Between-occupation differences in workforce composition and working conditions (Table 1) provide important context for interpreting the heterogeneous pathways. Dental hygienists had the highest mean emotional labor and burnout (Table 1), consistent with frequent and emotionally demanding patient-facing encounters. In contrast, job satisfaction did not differ substantially by occupation (Table 1), suggesting that differences in mean satisfaction alone do not explain burnout differences. Rather, the role of job satisfaction within the emotional labor–burnout pathway appears to be occupation-dependent (Table 4).

Supplementary fully adjusted regression results (Table S2) also suggested a non-linear association between length of service and burnout, with higher burnout observed in the 5–9 year group. This pattern may reflect a period in which role demands and responsibilities increase before stabilising with greater experience, underscoring the need for targeted supports for mid-career staff.

For physical therapists and dental hygienists, the indirect pathway via job satisfaction was not statistically supported (Table 4), whereas the direct association between emotional labor and burnout remained statistically supported. This pattern suggests that, for these occupations, interventions may need to focus more directly on reducing emotionally demanding interactions and improving immediate work conditions (e.g., staffing, scheduling, and recovery opportunities) rather than relying primarily on job satisfaction as the leverage point.

This manuscript extends prior analyses using the same K-VHS survey dataset [15] by addressing a distinct analytic objective. Whereas the prior report focused on describing occupational differences and occupation-stratified regression associations, the present study formally tested an occupation-specific moderated mediation mechanism using SEM and quantified conditional indirect effects as well as direct, indirect, and total associations by occupation (Tables 3 and 4; Fig. 1). We also provide complementary pooled interaction and marginal-effect results (Table 2; Table S1) and a fully adjusted regression model in the overall sample (Table S2) to support transparency and help readers triangulate findings across analytic approaches.In Korean healthcare settings, prior studies have highlighted the importance of organisational context for job satisfaction and burnout and the potential protective roles of social support and emotional competencies [27–29]. Such organisational resources may be particularly salient in public medical institutions, where staffing and service demands can differ from private hospitals [30].

The findings suggest that workforce interventions should be aligned with occupation-specific mechanisms. For clinical laboratory scientists and radiologic technologists, enhancing job satisfaction may be a plausible lever to mitigate burnout by reducing the indirect pathway from emotional labor to burnout (Table 4). For physical therapists and dental hygienists, interventions may need to focus more on reducing emotional labor burden and improving health and recovery supports, given the strong direct associations and the prominence of self-rated health status in supplementary analyses (Table S2). Across all occupations, the consistent positive direct association between emotional labor and burnout underscores the importance of organisational strategies that reduce emotional dissonance and strengthen protective resources, rather than relying solely on individual-level coping.

Several limitations warrant consideration. First, the cross-sectional design precludes causal inference; thus, indirect “effects” should be interpreted as statistical decomposition of associations rather than evidence of temporal mediation. Second, selection bias is possible because participation was voluntary and the response rate was moderate; a healthy-worker effect may have led to underestimation if workers with severe burnout were less likely to participate or had already left employment. and residual selection bias due to moderate response rate (50.9%) may have led to underestimation of burnout. Third, despite broadly standardised policies within the national system, residual hospital-level heterogeneity (e.g., leadership, workload intensity, and workplace climate) could confound associations. Fourth, all measures were self-reported, raising the possibility of reporting bias and common-method variance. Finally, the focus on four occupations within K-VHS hospitals may limit generalisability to other healthcare settings. Longitudinal and multi-setting studies are needed to clarify temporal ordering and to evaluate whether occupation-tailored interventions targeting emotional labor and job satisfaction reduce burnout.

Within the K-VHS system, emotional labor was consistently associated with higher burnout, and job satisfaction was inversely associated with burnout, but the pathway structure differed by occupation. Job satisfaction statistically accounted for part of the emotional labor–burnout association in clinical laboratory scientists and radiologic technologists but not in physical therapists or dental hygienists (Table 4). These results support occupation-tailored strategies that target the most relevant pathway for each group.

## Conclusions

Burnout was associated with emotional labor and job satisfaction, with the structure and magnitude of these associations differing across allied health occupations within the Korea Veterans Health Service. In structural equation modelling, job satisfaction statistically accounted for part of the emotional labor–burnout association in some occupations but not others, supporting occupation-tailored workforce strategies. Interventions may therefore need to combine approaches that reduce emotional labor demands with measures that strengthen job satisfaction-related resources, prioritised according to occupational profiles. Further longitudinal research is needed to clarify temporal ordering and to evaluate targeted interventions.

## Supplementary Information

Below is the link to the electronic supplementary material.

Supplementary Material 1

Supplementary Material 2: Table S1. Occupation-specific marginal effects of emotional labor and job satisfaction on burnout. Table S2. Multiple linear regression analysis of factors associated with burnout (N=290)

## Data Availability

The dataset analysed in this study was derived from an employee survey conducted across six Korea Veterans Health Service hospitals in July 2022. The data are not publicly available due to institutional restrictions and confidentiality considerations for participants, but may be made available from the corresponding author on reasonable request and with permission from the data-holding institution.
