# Supplementary material for: Emotional labor and burnout among healthcare workers in Korea: occupation-specific moderated mediation through job satisfaction (a cross-sectional secondary analysis)
Source: BMC Health Serv Res. 2026 Feb 7;26:351. doi: 10.1186/s12913-026-14166-1 (PMC12977388; doi:10.1186/s12913-026-14166-1)
Supplement: Supplementary file 1 — Supplementary Material 1 [file 12913_2026_14166_MOESM1_ESM.docx]

The following is an English translation of the survey, preserving the original formatting as much as possible:

The following are questions about general characteristics and work characteristics.

Please check the appropriate box.

1. What is your gender?

① Male ② Female

1. What is your age?

① 20s ② 30s ③ 40s ④ 50s ⑤ 60s or older

1. What is your marital status?

① Unmarried ② Married ③ Other

1. What is your height?

Ex) If you are 175cm tall, please enter 175 only.

1. What is your weight?

Ex) If you weigh 55kg, please enter 55 only

1. Do you have any hobbies or exercise that you do regularly?

① No ② Yes

1. What is your highest level of education?

① Junior college ② University ③ Graduate school or above

1. Do you have a religion?

① No ② Yes

1. Which hospital are you affiliated with?

① Central Veterans Hospital ② Gwangju Veterans Hospital ③ Busan Veterans Hospital ④ Daejeon Veterans Hospital ⑤ Daegu Veterans Hospital ⑥ Incheon Veterans Hospital

1. What is your job?

① Clinical Laboratory Scientist ② Radiological Technologist ③ Physical Therapist ④ Dental Hygienist

1. Does your job involve responding to patients?

① No ② Yes

1. What is your employment status?

① Regular ② Non-regular ③ Contract/Temporary/Part-time

1. Which of the following best describes your work schedule?

① Day shift ② Other (rotating, night)

1. How long have you been working at your current workplace?

① Less than 2 years ② 2-5 years ③ 5-10 years ④ 10-20 years ⑤ 20 years or more

1. What is your total work experience after obtaining your license?

① Less than 2 years ② 2-5 years ③ 5-10 years ④ 10-20 years ⑤ 20 years or more

1. What is your position?

① Staff ② Assistant Manager ③ Supervisor ④ Manager ⑤ General Manager or above

1. What is your salary? (after-tax)

① Less than 2 million won ② 2 million won - 3 million won ③ 3 million won - 4 million won ④ 4 million won - 5 million won ⑤ 5 million won - 6 million won ⑥ 6 million won or more

1. What is your average commute time?

① Less than 30 minutes ② Less than 1 hour ③ Less than 1 hour and 30 minutes ④ Less than 2 hours ⑤ 2 hours or more

1. Do you think your health has deteriorated compared to last year due to your current work?

① Strongly agree ② Agree ③ Neutral ④ Disagree ⑤ Strongly disagree

1. What was the most important reason for choosing your current workplace?

① Salary, work environment, and benefits ② Size and future prospects of the facility ③ Training system ④ Convenient transportation ⑤ Other:

1. If you were given the opportunity to change jobs in the future, which of the following would you prioritize?

① Salary, work environment, and benefits ② Size and future prospects of the facility ③ Training system ④ Convenient transportation ⑤ Other:

The following questions are related to emotional labor.

Please select the answer that is closest to your thoughts or feelings while working.

1. I make a conscious effort not to express negative emotions to patients

① Strongly agree ② Agree ③ Neutral ④ Disagree ⑤ Strongly disagree

1. When dealing with patients, I have no choice but to express my emotions as required by the hospital

① Strongly agree ② Agree ③ Neutral ④ Disagree ⑤ Strongly disagree

1. I hide my true feelings in the process of dealing with patients for work.

① Strongly agree ② Agree ③ Neutral ④ Disagree ⑤ Strongly disagree

1. Efforts to control emotions are necessary for routine work performance

① Strongly agree ② Agree ③ Neutral ④ Disagree ⑤ Strongly disagree

1. The emotions I feel when dealing with patients are different from the emotions I actually express

① Strongly agree ② Agree ③ Neutral ④ Disagree ⑤ Strongly disagree

1. I sometimes deal with aggressive or difficult patients

① Strongly agree ② Agree ③ Neutral ④ Disagree ⑤ Strongly disagree

1. I sometimes deal with patients who demand things that are beyond my ability or authority

① Strongly agree ② Agree ③ Neutral ④ Disagree ⑤ Strongly disagree

1. I feel humiliated when responding to patients

① Strongly agree ② Agree ③ Neutral ④ Disagree ⑤ Strongly disagree

1. I have difficulty performing my duties due to unreasonable or unreasonable demands from patients

① Strongly agree ② Agree ③ Neutral ④ Disagree ⑤ Strongly disagree

1. I feel upset when I have to hide my feelings from patients and not express them

① Strongly agree ② Agree ③ Neutral ④ Disagree ⑤ Strongly disagree

1. When responding to patients, my emotions feel like a commodity

① Strongly agree ② Agree ③ Neutral ④ Disagree ⑤ Strongly disagree

1. The difficult emotions I felt when responding to patients remain even after work

① Strongly agree ② Agree ③ Neutral ④ Disagree ⑤ Strongly disagree

1. I get hurt emotionally in the process of dealing with patients

① Strongly agree ② Agree ③ Neutral ④ Disagree ⑤ Strongly disagree

1. Even when I'm physically tired, I have to do my best for the patients, so it's emotionally draining

① Strongly agree ② Agree ③ Neutral ④ Disagree ⑤ Strongly disagree

1. I am monitored (CCTV, etc.) to see if I respond well to patients as required by the hospital

① Strongly agree ② Agree ③ Neutral ④ Disagree ⑤ Strongly disagree

1. Patient evaluations affect my performance evaluations or personnel evaluations

① Strongly agree ② Agree ③ Neutral ④ Disagree ⑤ Strongly disagree

1. When there is a problem with patient response, I am treated unfairly by the hospital even if it is not my fault

① Strongly agree ② Agree ③ Neutral ④ Disagree ⑤ Strongly disagree

1. When problems arise in the process of responding to patients, the hospital takes appropriate action

① Strongly agree ② Agree ③ Neutral ④ Disagree ⑤ Strongly disagree

1. There are official systems and procedures within the hospital to resolve and assist with problems that arise in the process of responding to patients

① Strongly agree ② Agree ③ Neutral ④ Disagree ⑤ Strongly disagree

1. The hospital allows me to be comforted for the emotional wounds I suffered in the process of responding to patients

① Strongly agree ② Agree ③ Neutral ④ Disagree ⑤ Strongly disagree

1. My supervisor helps me resolve problems that arise in the process of responding to patients

① Strongly agree ② Agree ③ Neutral ④ Disagree ⑤ Strongly disagree

1. My colleagues help me resolve problems that arise in the process of responding to patients

① Strongly agree ② Agree ③ Neutral ④ Disagree ⑤ Strongly disagree

1. There are guidelines or manuals (instructions, guides) in the hospital regarding patient response

① Strongly agree ② Agree ③ Neutral ④ Disagree ⑤ Strongly disagree

1. I am given the authority or autonomy to resolve patient requests

① Strongly agree ② Agree ③ Neutral ④ Disagree ⑤ Strongly disagree

The following questions are about job satisfaction

Please select the answer that is closest to your thoughts or feelings

1. Are you interested in your current job?

① Strongly agree ② Agree ③ Neutral ④ Disagree ⑤ Strongly disagree

1. Do you think others consider your current job important?

① Strongly agree ② Agree ③ Neutral ④ Disagree ⑤ Strongly disagree

1. Do you think your current job has potential and will help you develop?

① Strongly agree ② Agree ③ Neutral ④ Disagree ⑤ Strongly disagree

1. Do you think your knowledge and skills are being well utilized in your current job?

① Strongly agree ② Agree ③ Neutral ④ Disagree ⑤ Strongly disagree

1. Do you think your workload and working hours are appropriate for your abilities?

① Strongly agree ② Agree ③ Neutral ④ Disagree ⑤ Strongly disagree

1. Do you think your supervisor listens to your suggestions or complaints sincerely?

① Strongly agree ② Agree ③ Neutral ④ Disagree ⑤ Strongly disagree

1. Does your supervisor praise you when you do a good job?

① Strongly agree ② Agree ③ Neutral ④ Disagree ⑤ Strongly disagree

1. Does your supervisor allow you a lot of autonomy in your work?

① Strongly agree ② Agree ③ Neutral ④ Disagree ⑤ Strongly disagree

1. Does your supervisor help you with your work?

① Strongly agree ② Agree ③ Neutral ④ Disagree ⑤ Strongly disagree

1. Is your supervisor interested in your personal matters?

① Strongly agree ② Agree ③ Neutral ④ Disagree ⑤ Strongly disagree

1. Does your supervisor evaluate you fairly?

① Strongly agree ② Agree ③ Neutral ④ Disagree ⑤ Strongly disagree

1. Do you have a lot of respect for your supervisor?

① Strongly agree ② Agree ③ Neutral ④ Disagree ⑤ Strongly disagree

1. Do you expect that if your organization's profits increase, the organization's profits will be appropriately distributed through bonuses, etc.?

① Strongly agree ② Agree ③ Neutral ④ Disagree ⑤ Strongly disagree

1. Are you satisfied with the way your organization determines base salary, bonuses, and allowances?

① Strongly agree ② Agree ③ Neutral ④ Disagree ⑤ Strongly disagree

Continuing the translation:

1. Are you satisfied with the way your organization determines promotions and transfers?

① Strongly agree ② Agree ③ Neutral ④ Disagree ⑤ Strongly disagree

1. Are you satisfied with the way your organization determines training opportunities?

① Strongly agree ② Agree ③ Neutral ④ Disagree ⑤ Strongly disagree

1. Do you think your organization provides you with sufficient opportunities for development?

① Strongly agree ② Agree ③ Neutral ④ Disagree ⑤ Strongly disagree

1. Do you think your organization's work environment is pleasant?

① Strongly agree ② Agree ③ Neutral ④ Disagree ⑤ Strongly disagree

1. Do you think your organization's work environment is safe?

① Strongly agree ② Agree ③ Neutral ④ Disagree ⑤ Strongly disagree

1. Do you think the equipment and tools provided by your organization are sufficient to perform your job?

① Strongly agree ② Agree ③ Neutral ④ Disagree ⑤ Strongly disagree

1. Do you think your organization provides you with enough rest time?

① Strongly agree ② Agree ③ Neutral ④ Disagree ⑤ Strongly disagree

1. Do you think your organization provides you with enough welfare benefits?

① Strongly agree ② Agree ③ Neutral ④ Disagree ⑤ Strongly disagree

1. Do you think your organization's personnel system is fair?

① Strongly agree ② Agree ③ Neutral ④ Disagree ⑤ Strongly disagree

1. Do you think your colleagues are friendly and helpful?

① Strongly agree ② Agree ③ Neutral ④ Disagree ⑤ Strongly disagree

1. Do you think your colleagues are competent?

① Strongly agree ② Agree ③ Neutral ④ Disagree ⑤ Strongly disagree

1. Do you think your organization's communication with employees is smooth?

① Strongly agree ② Agree ③ Neutral ④ Disagree ⑤ Strongly disagree

1. Do you think your organization's decision-making process is transparent?

① Strongly agree ② Agree ③ Neutral ④ Disagree ⑤ Strongly disagree

1. Overall, are you satisfied with your job?

① Strongly agree ② Agree ③ Neutral ④ Disagree ⑤ Strongly disagree

1. If you were given the opportunity, would you recommend your current workplace to others?

① Strongly agree ② Agree ③ Neutral ④ Disagree ⑤ Strongly disagree

1. Do you intend to continue working at your current workplace?

① Strongly agree ② Agree ③ Neutral ④ Disagree ⑤ Strongly disagree

The following questions pertain to turnover intention.

Please select the answer that best aligns with your thoughts.

1. I often think about quitting my current job

① Strongly agree ② Agree ③ Neutral ④ Disagree ⑤ Strongly disagree

1. If I had the opportunity, I would move to another workplace

① Strongly agree ② Agree ③ Neutral ④ Disagree ⑤ Strongly disagree

1. I will continue to work at my current workplace even if the working conditions are poor

① Strongly agree ② Agree ③ Neutral ④ Disagree ⑤ Strongly disagree

1. I will leave my current workplace if I am offered a better job

① Strongly agree ② Agree ③ Neutral ④ Disagree ⑤ Strongly disagree

1. I am constantly looking for other jobs

① Strongly agree ② Agree ③ Neutral ④ Disagree ⑤ Strongly disagree

1. If I find a suitable job, I will leave my current job immediately

① Strongly agree ② Agree ③ Neutral ④ Disagree ⑤ Strongly disagree

1. I am willing to put in a lot of effort beyond what is normally expected to contribute to the success of this organization

① Strongly agree ② Agree ③ Neutral ④ Disagree ⑤ Strongly disagree

The following questions are about your organizational commitment.

Please select the answer that best aligns with your thoughts

1. I talk up this organization to my friends as a great organization to work for

① Strongly agree ② Agree ③ Neutral ④ Disagree ⑤ Strongly disagree

1. I feel very little loyalty to this organization

① Strongly agree ② Agree ③ Neutral ④ Disagree ⑤ Strongly disagree

1. I would accept almost any type of job assignment in order to keep working for this organization

① Strongly agree ② Agree ③ Neutral ④ Disagree ⑤ Strongly disagree

1. I find that my values and the organization’s values are very similar

① Strongly agree ② Agree ③ Neutral ④ Disagree ⑤ Strongly disagree

1. I am proud to tell others that I am part of this organization

① Strongly agree ② Agree ③ Neutral ④ Disagree ⑤ Strongly disagree

1. I could just as well be working for a different organization as long as the type of work were similar

① Strongly agree ② Agree ③ Neutral ④ Disagree ⑤ Strongly disagree

1. This organization really inspires the very best in me in the way of job performance

① Strongly agree ② Agree ③ Neutral ④ Disagree ⑤ Strongly disagree

1. It would take very little change in my present circumstances to cause me to leave this organization

① Strongly agree ② Agree ③ Neutral ④ Disagree ⑤ Strongly disagree

1. I am extremely glad that I chose this organization to work for over others I was considering at the time I joined

① Strongly agree ② Agree ③ Neutral ④ Disagree ⑤ Strongly disagree

1. There’s not much to be gained by sticking with this organization indefinitely

① Strongly agree ② Agree ③ Neutral ④ Disagree ⑤ Strongly disagree

1. For me this is the best of all possible organizations for which to work

① Strongly agree ② Agree ③ Neutral ④ Disagree ⑤ Strongly disagree

1. I feel that my workload is excessive.

① Strongly agree ② Agree ③ Neutral ④ Disagree ⑤ Strongly disagree

1. I don't have enough time to perform my duties.

① Strongly agree ② Agree ③ Neutral ④ Disagree ⑤ Strongly disagree

1. I lack the skills or knowledge necessary to perform my duties.

① Strongly agree ② Agree ③ Neutral ④ Disagree ⑤ Strongly disagree

1. I lack the authority necessary to perform my duties.

① Strongly agree ② Agree ③ Neutral ④ Disagree ⑤ Strongly disagree

1. I lack the information or materials necessary to perform my duties

① Strongly agree ② Agree ③ Neutral ④ Disagree ⑤ Strongly disagree

1. I lack the manpower or equipment necessary to perform my duties

① Strongly agree ② Agree ③ Neutral ④ Disagree ⑤ Strongly disagree

1. My work objectives are unclear

① Strongly agree ② Agree ③ Neutral ④ Disagree ⑤ Strongly disagree

1. I receive conflicting demands at the same time while performing my duties

① Strongly agree ② Agree ③ Neutral ④ Disagree ⑤ Strongly disagree

1. I feel conflicted between conflicting demands while performing my duties

① Strongly agree ② Agree ③ Neutral ④ Disagree ⑤ Strongly disagree

1. I lack the ability to perform my duties

① Strongly agree ② Agree ③ Neutral ④ Disagree ⑤ Strongly disagree

Thank you for your participation in the survey.
