# Supplementary material for: Emotional labor and burnout among healthcare workers in Korea: occupation-specific moderated mediation through job satisfaction (a cross-sectional secondary analysis)
Source: BMC Health Serv Res. 2026 Feb 7;26:351. doi: 10.1186/s12913-026-14166-1 (PMC12977388; doi:10.1186/s12913-026-14166-1)
Supplement: Supplementary file 2 — Supplementary Material 2: Table S1. Occupation-specific marginal effects of emotional labor and job satisfaction on burnout. Table S2. Multiple linear regression analysis of factors associated with burnout (N=290) [file 12913_2026_14166_MOESM2_ESM.docx]

Table S1. Occupation-specific marginal effects of emotional labour and job satisfaction on burnout

| Occupation | dydx(c_emo) | *p* | 95% CI | dydx(c_duty) | *p* | 95% CI |
| --- | --- | --- | --- | --- | --- | --- |
| 1 Clinical laboratory scientists | 0.26 | 0.010 | 0.06 to 0.45 | -0.14 | 0.012 | -0.24 to -0.03 |
| 2 Radiologic technologists | 0.23 | 0.061 | -0.01 to 0.48 | -0.25 | 0.001 | -0.38 to -0.11 |
| 3 Physical therapists | 0.37 | <0.001 | 0.22 to 0.51 | -0.11 | 0.059 | -0.22 to 0.00 |
| 4 Dental hygienists | 0.55 | <0.001 | 0.26 to 0.84 | -0.02 | 0.769 | -0.18 to 0.14 |

These are within-occupation slopes from the pooled interaction model. Between-occupation differences were not significant in formal interaction tests.

**Table S2.** Multiple linear regression analysis of factors associated with burnout (N=290)

| Variables | B | t | *p* |
| --- | --- | --- | --- |
| Emotional labour | 0.362 | 7.310 | <0.001 |
| Job satisfaction | -0.124 | -3.820 | <0.001 |
| sex |  |  |  |
| Female | 3.241 | 2.660 | 0.008 |
| age |  |  |  |
| 30–39 | -2.938 | -1.760 | 0.080 |
| 40–49 | -5.067 | -1.950 | 0.052 |
| 50–60 | -2.826 | -0.830 | 0.410 |
| married |  |  |  |
| Married | -0.517 | -0.370 | 0.713 |
| Unmarried | 3.153 | 0.540 | 0.589 |
| BMI |  |  |  |
| normal (18.5-22.9) | -1.043 | -0.500 | 0.617 |
| overweight (≥23) | 1.169 | 0.520 | 0.606 |
| hobbies and exercise |  |  |  |
| Yes | -1.382 | -1.230 | 0.220 |
| Education |  |  |  |
| University | -1.254 | -1.060 | 0.291 |
| Graduate school or higher | -3.574 | -2.020 | 0.045 |
| religion |  |  |  |
| Yes | -0.021 | -0.020 | 0.984 |
| Customer service |  |  |  |
| Yes | 0.336 | 0.240 | 0.813 |
| employment status (employ) |  |  |  |
| Permanent contract | -18.779 | -2.200 | 0.029 |
| Temporary/contract | 0.248 | 0.120 | 0.908 |
| Part-time | -3.865 | -0.860 | 0.390 |
| work pattern |  |  |  |
| Day and night | -0.396 | -0.240 | 0.811 |
| Service period (year) |  |  |  |
| 2–4 | 3.515 | 1.750 | 0.082 |
| 5–9 | 5.295 | 2.240 | 0.026 |
| 10–19 | 4.554 | 1.690 | 0.091 |
| ≥20 | 3.550 | 0.970 | 0.335 |
| position (position) |  |  |  |
| Assistant manager | -2.488 | -1.820 | 0.070 |
| Senior staff | -3.752 | -1.550 | 0.122 |
| Manager | -4.111 | -1.530 | 0.126 |
| General manager | -4.809 | -1.290 | 0.197 |
| Pay |  |  |  |
| 200–299 | -1.629 | -0.500 | 0.617 |
| 300–399 | -1.899 | -0.560 | 0.578 |
| 400–499 | -2.672 | -0.650 | 0.518 |
| 500–599 | -0.439 | -0.090 | 0.928 |
| 600≤ | -4.017 | -0.830 | 0.410 |
| weekly working hours |  |  |  |
| <1 | -1.990 | -1.800 | 0.073 |
| <1.5 | -2.767 | -1.550 | 0.122 |
| <2 | -13.312 | -2.210 | 0.028 |
| ≥2 | -4.049 | -0.960 | 0.339 |
| self-rated health status |  |  |  |
| Poor | 10.005 | 3.950 | <0.001 |
| Moderate | 11.500 | 4.570 | <0.001 |
| Good | 14.043 | 5.300 | <0.001 |
| Very good | 15.535 | 5.040 | <0.001 |
| Constant | 39.059 | 4.460 | <0.001 |

Abbreviations: BMI, body mass index.
